# *Cellulomonas endometrii* sp. nov.: a novel bacterium isolated from the endometrial microbiota

**DOI:** 10.1007/s00203-023-03703-9

**Published:** 2023-11-08

**Authors:** Linda Abou Chacra, Marion Bonnet, Mégane Heredia, Gabriel Haddad, Nicholas Armstrong, Stéphane Alibar, Florence Bretelle, Florence Fenollar

**Affiliations:** 1 Aix-Marseille Université, IRD, AP-HM, SSA, VITROME, Marseille, France; 2 IHU-Méditerranée Infection, Marseille, France; 3 Aix-Marseille Université, IRD, AP-HM, MEPHI, Marseille, France; 4 Department of Gynaecology and Obstetrics, Gynépole, La Conception, AP-HM, Marseille, France

**Keywords:** *Cellulomonas endometrii* sp. nov., Anaerobic, New bacterial species, Endometrial microbiota, Endometritis, Human microbiota, Taxonomy

## Abstract

An isolate of a bacterium recovered from an endometrial biopsy failed to be identified by MALDI-TOF mass spectrometry and was subjected to 16S rRNA sequencing. The obtained sequence was compared by BLASTn against the NCBI database, which revealed that the most closely related species was *Cellulomonas hominis* and *Cellulomonas pakistanensis*, with 98.85% and 98.45% identity, respectively. Phenotypic characterisation and genome sequencing were performed. The isolate was facultative anaerobic, gram-positive, motile, non-spore forming, and rod-shaped. Cell wall fatty acid profiling revealed that 12-methyl-tetradecanoic acid was the most abundant fatty acid (36%). The genome size was 4.25 Mbp with a G + C content of 74.8 mol%. Genomic comparison of species closely related to this strain showed that all digital DNA–DNA hybridisation (dDDH) and mean orthologous nucleotide identity (OrthoANI) values were below published species thresholds (70% and 95–96%, respectively). Based on these data, we conclude that this isolate represents a new bacterial species belonging to the family *Cellulomonadaceae* and the phylum *Actinomycetota.* We propose the name *Cellulomonas endometrii* sp. nov. The type strain is Marseille-Q7820^T^ (= CSUR Q7820 = CECT 30716).

**Supplementary Information:**

The online version contains supplementary material available at 10.1007/s00203-023-03703-9.

## Introduction

The endometrial microbiota was recently discovered and explored. It inhabits the lining of the uterus, previously thought to be sterile (Moreno and Franasiak 2017; Toson et al. 2022). Emerging evidence suggests that the endometrial microbiota plays an important role in reproductive health and may have implications for conditions, such as infertility, endometriosis, and miscarriage (Moreno et al. 2016; Toson et al. 2022). The composition and diversity of the endometrial microbiota have been found to vary significantly between women and may be influenced by factors, such as hormonal fluctuations, the menstrual cycle, and pregnancy (Benner et al. 2018; Toson et al. 2022).

However, much is still unknown about the endometrial microbiota and how it interacts with the host immune system and reproductive processes (Benner et al. 2018). Further research is needed to fully understand the role of the endometrial microbiota in health and disease and to develop potential diagnostic and therapeutic interventions (Toson et al. 2022). The emergence of culturomics, a powerful tool to study microbial diversity, has enabled the isolation and characterisation of many new bacterial species from various microbiota; however, this culture strategy had never been performed from an endometrial biopsy (Lagier et al. 2015; Lagier and Raoult 2016).

By applying this method, we succeeded in isolating a new member of the genus *Cellulomonas* from an endometrial biopsy designated Marseille-Q7820. Using a polyphasic taxonogenomic approach which integrates the annotated whole genome, proteomic information obtained from MALDI-TOF MS spectra, and phenotypic features, we provide a detailed description of this strain in pure culture.

## Materials and methods

### Ethical approval, sampling, and isolation of the strain

An endometrial biopsy sampled for diagnostic purposes was sent to the clinical microbiology laboratory of Marseille Public University Hospitals (AP-HM, France) where it was retrospectively analysed using a culturomics strategy, as permitted by French law (Article L.1211-2 of the French Code on Public Health). The patient was informed of the possible reuse for research purposes of her sample and the personal data collected from her during her care. She could oppose this by reporting it to the data protection officer at the AP-HM. The patient did not express any objection. The personal data that were used for analysis were anonymised. Our independent local ethics committee (Agreement No. 2022-009) approved the clearance of ERC and compliance with data protection legislation.

Strain Marseille-Q7820 was thus obtained from an endometrial biopsy sample taken from a 34-year-old woman who had suffered from recurrent early miscarriage and chronic endometritis. The vaginal sample collected during the consultation revealed no bacterial vaginosis or sexually transmitted infections. The endometrial biopsy was performed after cleaning the cervix and vagina with sterile compresses soaked in Dakin®. The endometrial biopsy catheter tip (Pipelle^®^, CDD laboratory, Paris, France) was inserted through the cervix into the uterine cavity to avoid contamination from the nearby tissues. The biopsy was then aspirated by suction.

To isolate strain Marseille-Q7820, the endometrial biopsy was pre-incubated in anaerobic blood culture vials (Becton Dickinson, Le Pont-de-Claix, France) supplemented with Difco Marine Broth (Becton Dickinson) at 37 °C for 1 day. Isolated colonies were then obtained through subculture on Columbia agar with 5% sheep blood (bioMérieux, Marcy l’Etoile, France) and were incubated at 37 °C in anaerobic conditions using AnaeroGen (bioMérieux) for 48 h.

### MALDI-TOF identification, 16S rRNA identification, and phylogenetic analysis

Strain Marseille-Q7820 was identified using a Microflex LT MALDI-TOF mass spectrometer (Bruker Daltonics, Bremen, Germany) following the protocol described by Seng et al*.* (2013). The MALDI BioTyper software (version 2.0, Bruker) was used to analyse the spectra of the strain by standard pattern matching with default parameter settings. The resulting scores were interpreted as previously described by Hadjadj et al*.* (2016).

### Morphological observation, phenotypic characterisation, and antibiotic susceptibility

The optimal growth conditions for the Marseille-Q7820 strain were determined by subjecting it to different atmospheric, temperature, pH, and salinity parameters. Thus, the strain was cultured on Columbia agar with 5% sheep blood (bioMérieux) under different atmospheric conditions, in particular aerobic, anaerobic (GENbag anaer, bioMérieux), and microaerophilic (GENbag Microaer) conditions, at different temperatures, including 10 °C, 20 °C, 28 °C, 37 °C, 42 °C, and 56 °C. The pH levels used were 5.5, 6, 6.5, 7, 7.5, 8, and 8.5; NaOH or HCl buffers were added to adjust the desired pH of the culture medium. The salinity levels varied within the range of 0.5%, 5%, 7.5%, 10%, 15%, and 20%. Additionally, the Marseille-Q7820 strain was subjected to various tests to determine the phenotypic characteristics, including Gram staining, motility, sporulation, and oxidase and catalase activities, as reported previously (Ly et al. 2022). The morphology was examined using an SU5000 scanning electron microscope (SEM, Hitachi High-Technologies, Tokyo, Japan), as presented by Zgheib et al*.* (2021). The strain’s biochemical properties were assessed using three API gallery systems (API^®^ ZYM, API^®^ 20A, and API^®^ 50 CH [bioMérieux]) according to the manufacturer’s instructions. The analysis of cellular fatty acid methyl esters (FAME) was conducted using gas chromatography/mass spectrometry (GC/MS) according to the methods previously described (Dione et al. 2016; Sasser 2006). Finally, antimicrobial susceptibility testing was assessed using E-test gradient strips (bioMérieux) in compliance with EUCAST recommendations (Matuschek et al. 2014).

### Genome extraction, sequencing, annotation, and comparison

Strain Marseille-Q7820 was subjected to mechanical treatment with acid-washed glass beads (G4649-500 g, Sigma-Aldrich, Saint-Quentin-Fallavier, France) using a FastPrep BIO 101 instrument (Qbiogene, Strasbourg, France) at maximum speed (6.5 m/s) for 90 s, followed by a lysozyme incubation for two hours at 37 °C. DNA was then extracted using the EZ1 Robot and the EZ1 DNA Tissue kit (Qiagen, Hilden, Germany). The DNA extracted was subjected to sequencing using a MiSeq sequencer (Illumina Inc., San Diego, CA, USA) and the Nextera Mate Pair sample preparation kit, along with the Nextera XT Paired End preparation kit (Illumina), following the methods previously outlined in Anani et al*.* (2019).

The resulting reads were assembled using SPAdes 3.13.1 software, excluding scaffolds below 800 bp and depth values below 25% of the average depths. The obtained genome was annotated using Prokka 1.14.5 (Seemann 2014; Zgheib et al. 2020) and compared with those of closely related species.

The 16S rRNA sequence was also analysed. The 16S rRNA sequences of the Marseille-Q7820 strain and their closely related species were aligned, and a phylogenetic tree was constructed with 1000 bootstrap replicates, based on the Neighbor-Joining method (Saitou and Nei 1987) and the Kimura 2-parameter methods (Kimura 1980), using the MEGA X software (Kumar et al. 2018).

Overall similarity among the genomes was evaluated using digital DNA–DNA hybridisation (dDDH) with the Genome-to-Genome Distance Calculator (GGDC) 2.1 web server (http://ggdc.dsmz.de/distcalc2.php) and mean nucleotide identity analysis with OrthoANI 1.2 software (Lee et al. 2016), respectively. The genome-based phylogenetic tree was automatically generated using TYGS for the Marseille-Q7820 strain and closely related species. The tree was deduced using FastME from GBDP distances calculated from genomic sequences.

## Results

### Strain identification and phylogenetic analysis

Strain Marseille-Q7820 was isolated from an endometrial biopsy. After performing a comprehensive analysis using MALDI-TOF mass spectrometry, we failed to identify the isolate. The score obtained was less than 1.8, indicating that the species was not in the database and could potentially belong to an unknown species.

The 16S rRNA sequence of the Marseille-Q7820 strain (OX458243.1) revealed 98.85% similarity to *Cellulomonas hominis* strain CE40 (NR_029288.1) and 98.45% to *Cellulomonas pakistanensis* strain NCCP-11 (NR_125452.1), the closest phylogenetically related species with standing in nomenclature.

**Fig. 1:**
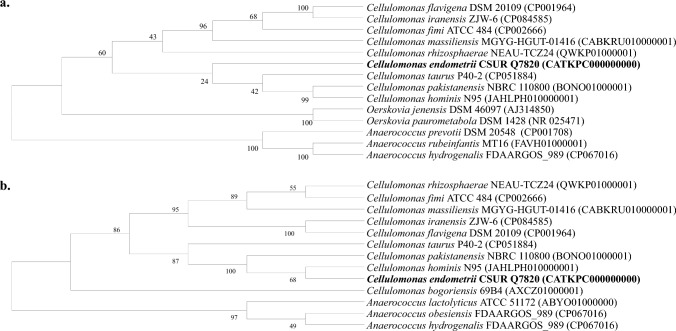
**a** 16S rRNA-based phylogenetic tree of *Cellulomonas endometrii* sp. nov., strain Marseille-Q7820 (bold) and closely related species. **b** Whole genome-based phylogenetic tree of *Cellulomonas endometrii* sp. nov., strain Marseille-Q7820 (bold) and closely related species. Accession numbers of the genomes used for the comparison are indicated in parentheses

The phylogenetic tree in Fig. 1 presents the position of strain Marseillle-Q7820 in relation to other closely related species with a validly published name.

### Phenotypic characterisation

**Fig. 2:**
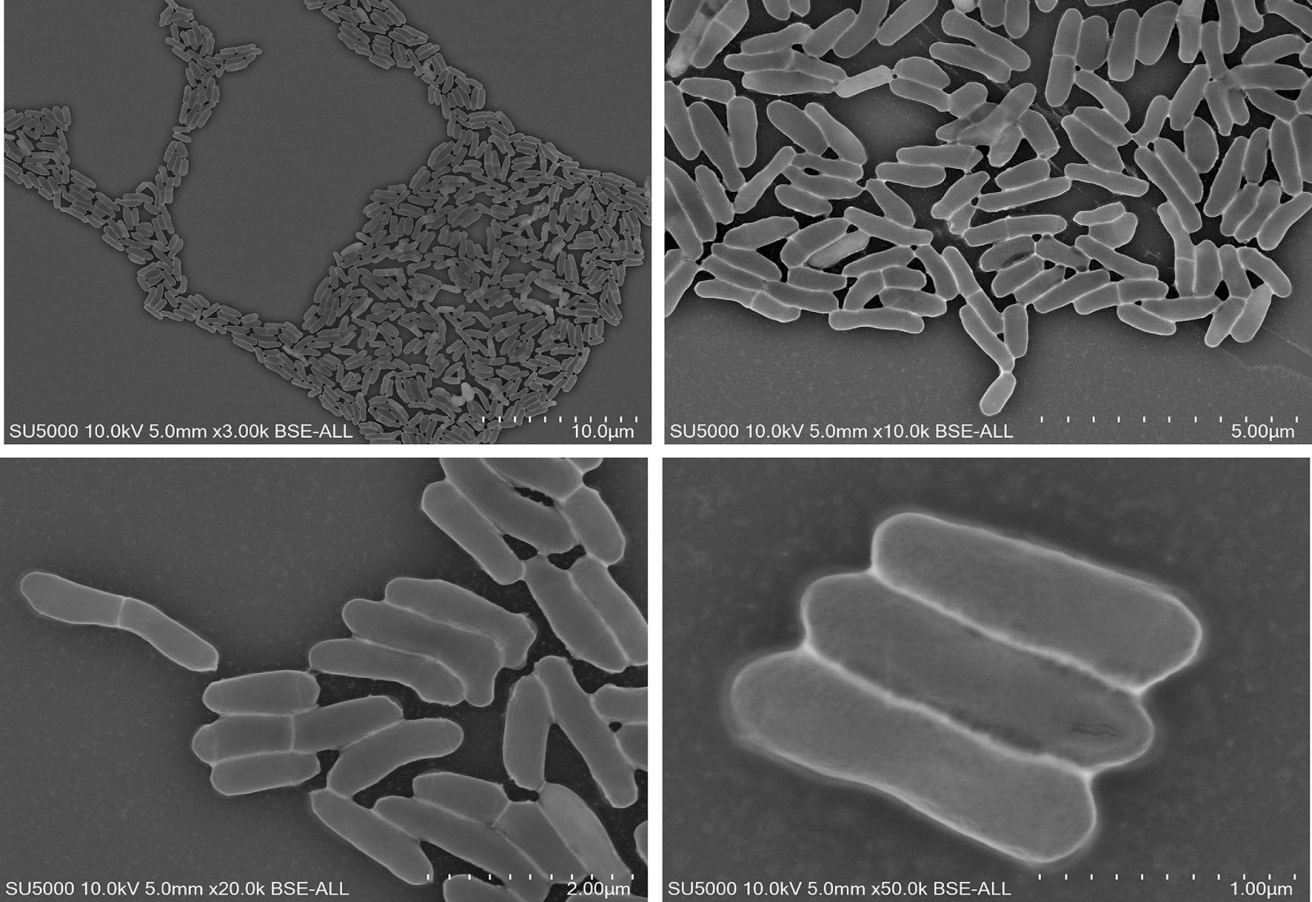
Microscopy image of *Cellulomonas endometrii* sp. nov., strain Marseille-Q7820. The image was obtained using an SU5000 SEM

The main characteristics of the Marseille-Q7820 strain are summarised in Supplementary Table 1. The Marseille-Q7820 strain showed optimal growth when cultured under anaerobic conditions at 37 °C for 2 days. Under these culture conditions, colonies are circular, pale yellow, opaque, and convex with an average diameter of 2 mm. Growth was also observed for the Marseille-Q7820 strain in a microoxic atmosphere. The Marseille-Q7820 strain is a gram-positive, motile (sliding), non-spore forming, and rod-shaped bacterium, positive for catalase and negative for oxidase. Using scanning electron microscopy, strain Marseille-Q7820 was determined to have an average diameter of 0.474 μm ± 0.075 μm and a length of 1.546 μm ± 0.283 μm (Fig. 2).

Using an API ZYM strip, positive results were obtained for leucine arylamidase, naphtol-AS-BI-phosphohydrolase, ß-galactosidase, D-glucosidase, ß-glucosidase, and N-acetyl-ß-glucosaminidase. Using an API 20A strip, positive reactions were observed for D-glucose, D-maltose, salicin, D-xylose, L-arabinose, gelatine, esculin, D-cellobiose, D-trehalose, and D-mannitol. Using an API 50 CH strip, a positive result was shown for L-arabinose, D-xylose, methyl-xyloside, galactose, D-glucose, D-fructose, D-mannose, mannitol, methyl-D-mannoside, N-acetyl-glucosamine, amygdalin, arbutin, esculin, salicin, cellobiose, maltose, sucrose, trehalose, gentibiose, turanose, gluconate, erythritol, rhamnose, dulcitol, sorbitol, methyl-D-glucoside, lactose, D-raffinose, xylitol, lysose, tagatose, and 5-keto-gluconate. These results were compared with those of *Cellulomonas pakistanensis* DSM 24792*, Cellulomonas hominis* DSM 9581, *Cellulomonas flavigena* JCM 18109 (Ahmed et al. 2014), and *Cellulomonas taurus* strain P40-2 (Zhang et al. 2021) (Table 1). The most abundant fatty acid for the Marseille-Q7820 strain was 12-methyl-tetradecanoic acid (41.8%), followed by hexadecanoic acid (29.3%) and 14-methyl-hexadecanoic acid (6%). Small amounts of unsaturated, branched, and saturated fatty acids were also described. This profile was similar to those described for the closest strains (Table 2).

**Table 1:** Comparison of strains *Cellulomonas endometrii* Marseille-Q7820, *Cellulomonas pakistanensis* DSM 24792*, Cellulomonas hominis* DSM 9581, *Cellulomonas flavigena* JCM 18109, and *Cellulomonas taurus* P40-2

Properties	*C. endometrii* Marseille-Q7820*	*C. pakistanensis* DSM 24792*	*C. hominis* DSM 9581*	*C. flavigena* JCM 18109**	*C. taurus* P40-2**
0_2_ requirement	Facultative anaerobic	Facultative anaerobic	Facultative anaerobic	Strictly aerobic	Facultative anaerobic
Gram stain	+	+	+	+	+
Mobility (Sliding)	+	+	+	–	+
Catalase	+	+	+	+	+
Oxidase	–	–	–	–	–
Production of					
Naphthol-AS-BI phosphohydrolase	+	w	+	w	NA
Valine arylamidase	–	–	–	–	NA
α-glucosidase	+	+	+	+	+
ß-glucosidase	+	+	+	w	+
ß-glucuronidase	–	–	–	–	NA
ß-galactosidase	+	+	w	–	+
Lactose	+	–	–	–	–
D-Sorbitol	+	–	–	–	–
Amygdalin	+	w	+	–	+
D-Raffinose	+	+	–	–	–
L-Rhamnose	+	–	–	–	+
L-Fucose	+	–	–	–	+
D-Tagatose	+	–	–	–	+
N-Acetylglucosamine	+	–	w	–	–
Utilization of					
D-glucose	+	+	+	+	+
D-sucrose	–	+	+	+	NA
D-mannose	–	+	+	w	+
G + C content (mol%)	74.8	76.2	75.5	75.3	71.99
Habitat	Human endometrium	Rice grain	Spinal fluid	Livestock	Livestock

+  Positive, − negative, *w* weakly positive, *NA* not available

*Data obtained in the course of this study

**Data based on the literature

**Table 2:** Cellular fatty acid composition (%) of strains *Cellulomonas endometrii* Marseille-Q7820, *Cellulomonas pakistanensis* DSM 24792*, Cellulomonas hominis* DSM 9581, *Cellulomonas flavigena* JCM 18109, and *Cellulomonas taurus* P40-2

Fatty acids	Name	*C. endometrii* Marseille-Q7820*	*C. pakistanensis* DSM 24792*	*C. hominis* DSM 9581*	*C. flavigena* JCM 18109 **	*C. taurus* P40-2**
C16:0	Hexadecanoic acid	29.3	15.9	11.5	15.7	10.9
C15:0 iso	13-methyl-tetradecanoic acid	1.9	2.2	2.2	1.6	4.3
C15:0 anteiso	12-methyl-tetradecanoic acid	41.8	52.6	56.7	32.2	54.6
C14:0 iso	12-methyl-tridecanoic acid	TR	TR	TR	10.8	ND
C15:1 anteiso	12-methyl-tetradecenoic acid	ND	TR	TR	11.3	5.5
C16: iso	14-methyl-pentadecanoic acid	1.4	1.8	1.7	10.9	4.7
C18:1n9	9-octadecenoic acid	5	2.8	3.9	ND	ND
C17:0 anteiso	14-methyl-hexadecanoic acid	6	8.6	10.2	1.4	11.1
C17:0 iso	15-methyl-hexadecanoic acid	TR	TR	TR	ND	ND
C14:0	Tetradecanoic acid	6	8	5.8	10.3	1.9
C18:2n6	9,12-octadecadienoic acid	4.8	TR	TR	ND	ND
C18:0	Octadecanoic acid	2.2	1.5	2.1	ND	ND
C15:0	Pentadecanoic acid	1.1	2.9	2.9	3.9	ND
C17:0	Heptadecanoic acid	TR	1	1.2	TR	ND

*TR* trace amounts < 1%, *Nd* Not detected

*Data obtained in the course of this study

**Data based on the literature

The minimum inhibitory concentration was 6.75 μg/L for penicillin G, 3 μg/L for amoxicillin, 2 μg/L for ceftriaxon, 0.038 μg/L for imipenem, 8 μg/L for ciprofloxacin, 1.5 μg/L for azithromycin, 8 μg/L for clindamycin, 6 μg/L for daptomycin, 0.023 μg/L for doxycycline, 12 μg/L for gentamicin, 48 μg/L for nitrofurantoin, 0.002 μg/L for rifampicin, 0.75 μg/L for linezolid, 0.38 μg/L for teicoplanin, and 0.125 μg/L for vancomycin. In addition, the Marseille-Q7820 strain was resistant to amikacin, tobramycin, fosfomycin, metronidazole, and trimethoprim-sulfamethoxazole.

### Genomic analysis

**Fig. 3:**
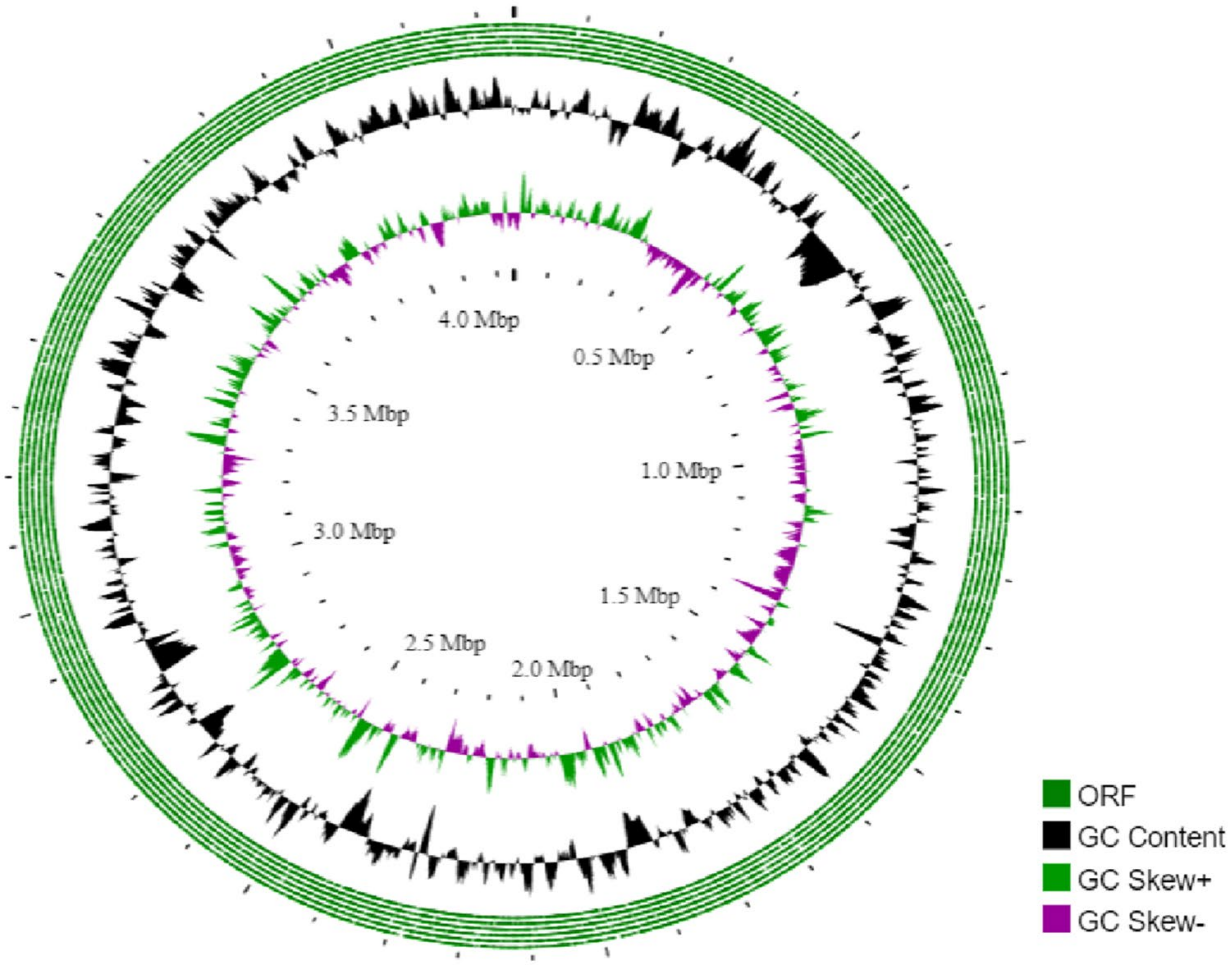
Genome map of *Cellulomonas endometrii* sp. nov., strain Marseille-Q7820 presented in a circular diagram

The Marseille-Q7820 strain exhibited a genome length of 4.25 Mbp, which was assembled into 39 contigs, with a G + C content of 74.8 mol% (Fig. 3). This strain was predicted to have 3922 genes, including 3864 protein-coding genes, as well as 58 RNA-coding genes, comprised of six rRNA, 51 tRNA, and one tmRNA (Table 3).

**Table 3:** Table summarising the genome properties of *Cellulomonas endometrii* sp. nov., strain Marseille-Q7820 and the other compared strains

Strains	Accession	Size (Mbp)	G + C (%)	Total genes	Protein-coding genes	rRNAs	tRNAs
*Cellulomonas endometrii* Marseille-Q7820	CATKPC000000000	4.25	74.8	3922	3864	6	51
*Cellulomonas hominis* N95	JAHLPH010000000	4.12	75.4	3813	3753	3	56
*Cellulomonas pakistanensis* NBRC 110800	BONO01000000	4.00	75.8	3678	3608	3	66
*Cellulomonas taurus* P40-2	CP051884	3.44	72.0	3177	3114	9	53

**Fig. 4:**
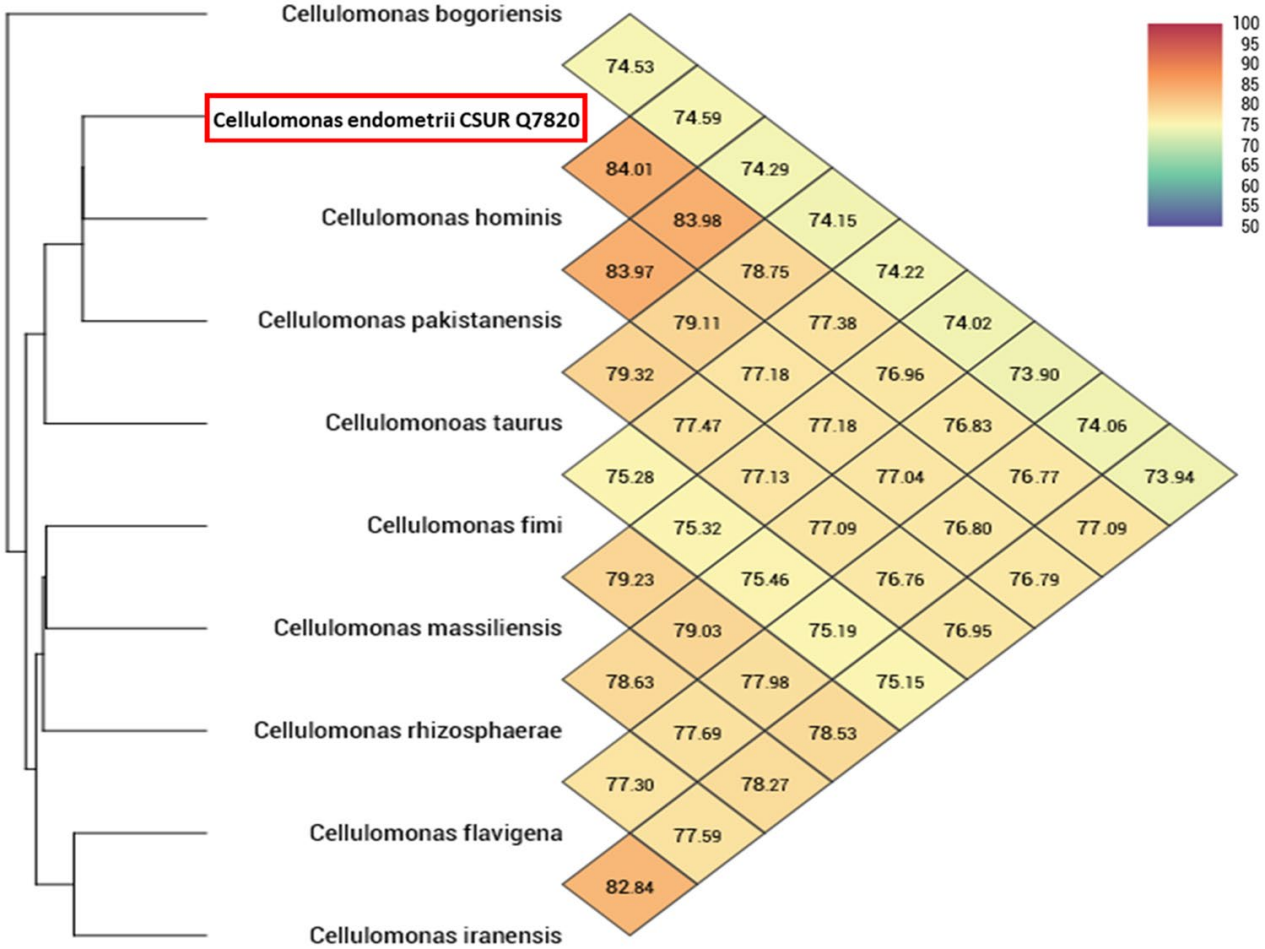
Heat map of OrthoANI values between *Cellulomonas endometrii* sp. nov., strain Marseille-Q7820 and closely related species. The values were calculated using OAT software and displayed as a colour gradient

Comparing the genomic features of this strain with other related species, the highest dDDH value obtained for the Marseille-Q7820 strain was 27.1% with *Cellulomonas hominis* (Table 4). Moreover, the OrthoANI values for strain Marseille-Q7820 ranged from 74.53 to 84.01%, further confirming its distinction from the other bacterial strains (Fig. 4).

**Table 4:** dDDH values of *Cellulomonas endometrii* sp. nov., strain Marseille-Q7820 with other closely related species with standing in nomenclature

Query strain	Subject strain	dDDH (in %)	G + C content difference (in %)
Q7820	*Cellulomonas hominis*	27.1	0.65
	*Cellulomonas pakistanensis*	26.9	1.01
	*Cellulomonas taurus*	21.6	2.77
	*Cellulomonas fimi*	21.3	0.04
	*Cellulomonas rhizosphaerae*	21.1	2.11
	*Cellulomonas flavigena*	21	0.47
	*Cellulomonas massiliensis*	21	0.08
	*Cellulomonas iranensis*	20.8	0.52
	*Cellulomonas bogoriensis*	20	2.53

## Conclusion

The 16S rRNA gene sequence identities between the strain Marseille-Q7820 and closely related species was 98.85% *Cellulomonas hominis*, 98.45% *C. pakistanensis*, and 98.16% *C. taurus*. Even if one of these values is higher than the 98.65% threshold commonly used for species delimitation (Kim et al. 2014; Stackebrandt and Goebel 1994), the strain is still considered a new bacterial species. Indeed, 16S rRNA gene sequences have shown their limitations in defining new bacterial species (Rossi-Tamisier et al. 2015).

Strain Marseille-Q7820 shows phenotypic differences from the closest species: (1) it does not use certain sugars, such as D-sucrose and D-mannose, unlike the others; (2) C18 fatty acids are also detected only in strain Marseille-Q7820. Additionally, dDDH and OrthoANI values between the compared genomes are also below established thresholds for species differentiation (70% and 95% to 96%, respectively) (Kim et al. 2014; Meier-Kolthoff et al. 2013).

Based on phenotypic, phylogenetic, and genomic analyses, we assert that strain Marseille-Q7820 is a new bacterium within the genera *Cellulomonas* in the family *Cellulomonadaceae* and the phylum *Actinomycetota.* Therefore, the name *Cellulomonas endometrii* sp. nov. is proposed.

Although almost 70 species of the genus *Cellulomonas* are listed, only 38 species have currently a validly published name under the List of Prokaryotic names with Standing in Nomenclature (https://lpsn.dsmz.de/search?word=Cellulomonas) (Parte et al. 2020). *Cellulomonas* spp. are mainly known as environmental pathogens, typically growing in decaying plant-rich soil, but they are also emerging rare human pathogens (Salas et al. 2014). Despite a number of isolates from human sources but of unknown clinical significance, bacteria of the genus *Cellulomonas* have only been isolated and implicated in active human infection in five cases in the literature to the best of our knowledge (Kimura et al. 2019).

Chronic endometritis is currently defined as a continuous and subtle inflammation characterised by the infiltration of plasma cells in the stromal zone of the endometrium (Kimura et al. 2019). Although the impact of chronic endometritis has long been ignored, because symptoms are either subtle or absent, the potential adverse effects of chronic endometritis on fertility have recently been shown (Kimura et al. 2019).

*Cellulomonas endometrii* sp. nov. was isolated from an endometrial biopsy from a woman with chronic endometritis and recurrent miscarriages. The first question that should be asked is whether this strain is a contaminant. As the strain was not isolated from the patient’s vaginal sample while being cultured in parallel with the same conditions, we can assume that *Cellulomonas endometrii* sp. nov. was part of the endometrial flora at the time of sampling. If this is the case, the other question that should be asked is whether this strain could potentially have a negative effect alone or in combination with other microorganisms. We do not currently have the elements to answer this, but the data underline the interest of exploring the endometrial microbiota by combining metagenomics and culturomics techniques.

### Description of *Cellulomonas endometrii* sp. nov.

*Cellulomonas endometrii* (en.do.me’tri.i. N.L. gen. neut. n. *endometrii*, from endometrium).

Facultative anaerobic, Gram-positive, non-spore forming, motile, and rod-shaped bacterium. Cells are 1.546 μm ± 0.283 μm in length and 0.474 μm ± 0.075 μm in diameter and positioned in clusters. Catalase activity is positive, while oxidase activity is negative. Colonies are visible on Columbia agar with 5% sheep blood incubated anaerobically after 2 days of growth. They appear circular, pale yellow, opaque, and convex with a diameter of 2 mm.

The type strain, Marseille-Q7820^T^, grows under anaerobic and microoxic conditions (optimally anaerobic) in temperatures ranging from 20 to 37 °C (optimally 37 °C), and requires a pH range of 6–8 (optimally pH 7) and a NaCl concentration of 0.5–15% (w/v) (optimally < 10%) for growth.

Using API strips, positive results were obtained for leucine arylamidase, naphtol-AS-BI-phosphohydrolase, ß-galactosidase, D-glucosidase, ß-glucosidase, N-acetyl-ß-glucosaminidase, D-glucose, D-maltose, salicin, D-xylose, L-arabinose, gelatine, esculin, D-cellobiose, D-trehalose, D-mannitol, methyl-xyloside, galactose, D-fructose, D-mannose, mannitol, methyl-D-mannoside, N-acetyl-glucosamine, amygdalin, arbutin, cellobiose, maltose, sucrose, trehalose, gentibiose, turanose, gluconate, erythritol, rhamnose, dulcitol, sorbitol, methyl-D-glucoside, lactose, D-raffinose, xylitol, lysose, tagatose, and 5-keto-gluconate.

The most abundant fatty acid by far was 12-methyl-tetradecanoic (41.8%), followed by hexadecanoic acid (29.3%) and 14-methyl-hexadecanoic acid (6%). The size of the genome is 4.25 Mbp and its G + C content is 74.8 mol%.

The type strain Marseille-Q7820^T^ (= CSUR Q7820 = CECT 30716) was isolated from an endometrial sample taken from a 34-year-old woman suffering from recurrent early miscarriage and chronic endometritis.

The 16S rRNA and genome sequences have been deposited in GenBank under accession numbers OX458243 and CATKPC000000000, respectively.

### Supplementary Information

Below is the link to the electronic supplementary material.

Supplementary file1 (DOCX 15 KB)

## Data Availability

The datasets presented in this search are available in online repositories. The names of the repository(s) and accession number(s) can be found below: https://www.ncbi.nlm.nih.gov/nuccore/CATKPC000000000. https://www.ncbi.nlm.nih.gov/nuccore/OX458243.
