# Supplementary material for: Cellulomonas endometrii sp. nov.: a novel bacterium isolated from the endometrial microbiota
Source: Arch Microbiol. 2023 Nov 8;205(12):377. doi: 10.1007/s00203-023-03703-9 (PMC10632280; doi:10.1007/s00203-023-03703-9)
Supplement: Supplementary file 1 — Supplementary file1 (DOCX 15 KB) [file 203_2023_3703_MOESM1_ESM.docx]

**Supplementary Table 1.** Main characteristics of *Cellulomonas endometrii* sp. nov., strain Marseille-Q7820.

| Properties | Strain Marseille-Q7820 |
| --- | --- |
| Genus name | *Cellulomonas* |
| Species name | *Cellulomonas endometrii* |
| Status | sp. nov. |
| Designation of the type strain | CSUR Q7820 |
| Strain collection numbers | CECT 30716 |
| 16S rRNA gene accession number | OX458243 |
| Genome accession number | CATKPC000000000 |
| Genome size | **4.25 Mbp** |
| G + C (mol %) | 74.8 |
| Origin | Marseille, France |
| Date of isolation | 2022 |
| Source of isolation | Human endometrial biopsy |
| Conditions used for standard cultivation | Columbia agar with 5% sheep blood after 48 h of incubation |
| Gram stain | Positive |
| Cell shape | Rod-shaped |
| Cell size | 1.546 ± 0.283 μm and 0.474 ± 0.075 μm |
| Motility | Motile |
| Sporulation | Non-sporulate |
| Colony morphology | Circular, pale yellow, opaque, and convex |
| Growth temperature | 20-37°C (37°C optimally) |
| Growth pH range | 6-8 (pH 7 optimally) |
| O_2_ requirement | Facultative anaerobic |
| Oxidase | Negative |
| Catalase | Positive |
| Growth salinity range | Tolerates up to 15% (0.5-10% optimally) |
